# Association of hyperactivity–impulsivity and inattention symptom profiles with suicide attempt: an 18-year population-based cohort study

**DOI:** 10.1136/bmjment-2025-301725

**Published:** 2025-07-11

**Authors:** Michel Spodenkiewicz, Ayla Inja, Samuele Cortese, Cedric Galera, Isabelle Ouellet-Morin, Sylvana M Côté, Michel Boivin, Frank Vitaro, Mara Brendgen, Ginette Dionne, Johanne Renaud, Richard E Tremblay, Gustavo Turecki, Marie-Claude Geoffroy, Massimilano Orri

**Affiliations:** 1 McGill Group for Suicide Studies, Douglas Mental Health University Institute, Department of Psychiatry, McGill University, Montreal, Quebec, Canada; 2 Pôle de Santé Mentale, CIC-EC 1410, CHU de La Réunion, Saint-Pierre, La Réunion, France; 3 Center for research in epidemiology and population health (CESP), National Institute of Health and Medical Research (INSERM) U1018, Paris-Saclay University, Villejuif, France; 4 Developmental EPI (Evidence synthesis, Prediction, Implementation) Lab, Centre for Innovation in Mental Health, School of Psychology, Faculty of Environmental and Life Sciences, University of Southamptopn, Southampton, UK; 5 Clinical and Experimental Sciences (CNS and Psychiatry), Faculty of Medicine, University of Southampton, Southampton, UK; 6 Hampshire and Isle of Wight Healthcare, NHS Foundation Trust, Southampton, UK; 7 Hassenfeld Children’s Hospital at NYU Langone, New York University Child Study Center, New York City, New York, USA; 8 DiMePRe-J-Department of Precision and Rigenerative Medicine-Jonic Area, University of Bari "Aldo Moro", Bari, Italy; 9 INSERM, Bordeaux Population Health Center, Bordeaux II University, Talence, France; 10 Centre Hospitalier Perrens, Bordeaux, France; 11 Department of Child and Adolescent Psychiatry, University of Bordeaux, Bordeaux, France; 12 Department of Criminology, Université de Montreal, Montreal, Quebec, Canada; 13 Research Centre of the Montreal Mental Health University Institute, Montreal, Quebec, Montreal; 14 Centre de recherche Azrieli, CHU Sainte-Justine, Montreal, Quebec, Canada; 15 Department of Social and Preventive Medicine, École de Santé Publique de l’Université de Montréal, Montreal, Quebec, Canada; 16 School of Psychology, Laval University, Québec City, Quebec, Canada; 17 School of Psychoeducation, University of Montreal, Montreal, Quebec, Canada; 18 Department of Psychology, Université du Québec à Montréal, Montreal, Quebec, Canada; 19 Department of Pediatrics and Psychology, University of Montreal, Montreal, Quebec, Canada; 20 Department of Epidemiology, Biostatistics, and Occupational Health, School of Population and Global Health, McGill University, Montreal, Quebec, Canada; 21 Danish Research Institute for Suicide Prevention, Mental Health Centre Copenhagen, Copenhagen, Denmark

**Keywords:** Child & adolescent psychiatry, Suicide & self-harm

## Abstract

**ABSTRACT:**

**Background:**

Attention-deficit/hyperactivity disorder (ADHD) symptomatology in childhood is associated with a high risk of suicide attempt later in life. However, symptom presentation in ADHD is heterogeneous, and little is known about how suicide risk varies according to different profiles of ADHD symptoms and sex.

**Objective:**

The aim was to investigate the longitudinal associations between childhood profiles of ADHD symptoms (ie, hyperactivity–impulsivity and inattention) and youth suicide attempt in males and females, separately.

**Methods:**

This population-based cohort study used data from three longitudinal cohorts: the Quebec Longitudinal Study of Child Development (QLSCD), the Quebec Longitudinal Study of Kindergarten Children (QLSKC) and the Quebec Newborn Twin Study (QNTS) for a total of 4399 participants (1490 from the QLSCD, 2134 from the QLSKC and 775 from the QNTS; 50% females) followed up from ages 6–23 years. Symptoms of hyperactivity–impulsivity and inattention were assessed by teachers five times from ages 6–12 years. Suicide attempt in adolescence and young adulthood (by age 23) was self-reported. Multitrajectory modelling was used to identify profiles of ADHD symptoms, and regression analysis was used to test their association with suicide attempt, adjusting for childhood socioeconomic and clinical characteristics.

**Findings:**

We identified four ADHD symptom profiles with distinct associations with suicide attempt for males and females. Compared with those with persistently low symptoms, females with persistently high inattention and hyperactivity–impulsivity (OR: 2.54, CI 1.39 to 4.63) or high inattention and low hyperactivity–impulsivity (OR: 1.81, CI 1.21 to 2.70) were at higher risk of suicide attempt, while, among males, only those with decreasing hyperactivity–impulsivity and inattention over time (OR: 2.23, CI 1.20 to 4.13) were at higher risk of suicide attempt.

**Conclusions:**

Risk of suicide attempt in children with ADHD symptoms varies according to both symptom profile and sex, the highest risk being for females with high inattention symptoms (with or without hyperactivity), and males with decreasing symptoms.

**Clinical implications:**

Taking into account differences in both sex and ADHD symptoms profile may be relevant to more accurately identify and manage suicide risk in individuals with high ADHD symptoms, though caution is needed when generalising our population-based findings to clinical populations.

**WHAT IS ALREADY KNOWN ON THIS TOPIC**

- Previous studies have shown that childhood attention-deficit/hyperactivity disorder (ADHD) symptomatology increases later risk of suicide attempt, but they did not account for ADHD symptoms heterogeneity and sex differences.

**WHAT THIS STUDY ADDS**

- By distinguishing between four symptom profiles of hyperactivity–impulsivity and inattention symptoms across childhood, this study showed that suicide risk in children with ADHD symptoms varies according to both symptom profile and sex, the highest risk being for females with high inattention symptoms (with and without high hyperactivity–impulsivity symptoms).

**HOW THIS STUDY MIGHT AFFECT RESEARCH, PRACTICE OR POLICY**

- Accounting for differences in both sex and symptom profile may be relevant to more accurately identify and manage suicide risk in individuals with ADHD.

## Background

 Attention-deficit/hyperactivity disorder (ADHD) is a common neurodevelopmental disorder, affecting 5%–7% of school-aged children.^1 2^ It is characterised by developmentally inappropriate and impairing levels of inattention and/or hyperactivity–impulsivity, usually emerging in early childhood.^1 2^ ADHD has been associated with poor physical and mental health, substance misuse as well as academic, relational and socioeconomic difficulties.^3 4^ Previous research also reported that children with ADHD are at increased risk of attempting suicide in adolescence.^5^,^8^ A meta-analysis has shown that, in cross-sectional and longitudinal studies, the risk of suicide attempt is more than twofold higher in children with high ADHD symptoms compared with their peers.^9^ Understanding the risk of suicide attempt among children with ADHD symptoms is therefore crucial to inform suicide prevention.

In this regard, it is crucial to consider two aspects of ADHD. First, ADHD is a heterogeneous condition from a clinical and neurobiological standpoint. Children with ADHD may exhibit predominantly hyperactivity–impulsivity symptoms, predominantly inattentive symptoms or a combination of both. Imaging and genetic studies have shown important brain network and genetic differences across such ADHD profiles.^10 11^ Furthermore, previous studies have shown that these ADHD symptom profiles were associated with distinct correlates and long-term outcomes. Children with combined hyperactivity–impulsivity and inattention symptoms had higher scores of externalising symptoms compared with children with other profiles, while children with predominantly inattentive symptoms had higher scores in internalising symptoms.^12^ Longitudinally, compared with children with high hyperactivity–impulsivity symptoms only, those with both inattentive and hyperactive-impulsive symptoms were more likely to meet criteria for externalising and bipolar disorders, while those with inattention symptoms only were more likely to meet criteria for major depression.^13^ Children with a profile predominantly characterised by inattention, rather than hyperactivity–impulsivity symptoms, were also more likely to have poorer educational outcomes.^14^ Yet, it is unclear if the risk of suicide attempt varies across distinct ADHD symptom profiles.^15^,^17^

Second, significant sex differences have also been reported in the ADHD symptoms presentation. Typically, males present with more hyperactivity symptoms than females, and females with more inattention symptoms than males.^18 19^ Females with ADHD symptoms are also more likely than males to experience comorbid internalising symptoms (eg, anxiety/depression) than males, while males are more likely to experience externalising symptoms (eg, conduct problems).^20^ It is thus important to consider sex differences in ADHD symptoms, especially when investigating associations with suicidal behaviour, which is two times more likely among females than males.^6^ Overall, given the substantial heterogeneity of ADHD, understanding the characteristics of individuals with ADHD symptoms who are at high risk of suicide attempts is critical to identify individuals at risk and inform clinical management programmes.

### Objective

Relying on longitudinal analyses of three population-based cohorts, the aim of this study was to investigate associations between distinct profiles of ADHD symptoms in childhood (ie, profiles characterised by different combinations of hyperactivity–impulsivity and inattention symptoms) and suicide attempt in adolescence and young adulthood. We specifically aimed to investigate males and females separately to highlight sex differences in the association between ADHD symptom profiles and suicide attempt. We hypothesised that suicide attempt risk differs both across ADHD symptom profiles and between sex, with profiles characterised by high inattention to be increasingly linked to suicide attempt in females than males.

## Methods

### Participants

We drew from three longitudinal population-based cohorts from the province of Quebec, Canada. The Quebec Longitudinal Study of Child Development (QLSCD) is a representative sample of 2120 infants born in 1997/1998.^21^ The original QLSCD sample was selected from the Quebec Birth Registry using a stratified procedure based on living area and birth rate, and data were collected yearly during childhood and biyearly during adolescence by the *Institut de la Statistique du Québec* (ISQ). The Longitudinal Study of Kindergarten Children (QLSKC) is a longitudinal cohort of 3017 children attending kindergarten in Quebec’s French-speaking public schools between 1986 and 1987.^22^ The QLSKC includes 2000 participants who were selected using a random sampling procedure stratified by administrative region, school board size and sex to be representative of the population, plus an additional sample of 1017 participants who exhibited disruptive behaviours in kindergarten. The Quebec Newborn Twin Study (QNTS) is an ongoing prospective longitudinal cohort of twins born between 1995 and 1998.^23^ Recruitment for QNTS was initiated with the Quebec Newborn Twin Registry, which identified all twin births in Quebec between 1995 and 1998, of which a total of 662 families of twins were initially assessed. We included in our study all individuals with available data on suicide attempt and for hyperactivity–impulsivity and inattention symptoms for at least one data point (online supplemental figure S1). We thus included in the study sample of 4399 participants (1490 from the QLSCD, 2134 from the QLSKC and 775 from the QNTS, respectively, 70.3%, 70.1% and 58.5% of the initial samples), with information on hyperactivity–impulsivity and inattention symptoms from 6 to 12 years of age and suicide attempt self-reported by youths themselves by age 23 years. We decided not to impute missing data on the outcome, but rather to select individuals based on outcome data availability.

### Measures

#### Teacher ratings of hyperactivity–impulsivity and inattention symptoms

For each cohort, behavioural ratings of hyperactivity–impulsivity and inattention were obtained from teacher reports at ages 6, 7, 8, 10 and 12 years. Symptoms in the previous 6 months were rated from five items of the Social Behavior Questionnaire (SBQ).^24^ Hyperactivity–impulsivity items were: ‘Can’t sit still’ and ‘is restless or hyperactive’. Inattention items were: ‘cannot pay attention for long’; ‘Is inattentive’ and ‘easily distracted’. Items were answered on a three-point Likert-type scale (never/not true, sometimes/somewhat true and often/very true). Hyperactivity–impulsivity and inattention scores were created by averaging the respective items at each time point. Cronbach’s alphas ranged from 0.78 to 0.90 for hyperactivity–impulsivity and from 0.83 to 0.90 for inattention.

#### Self-reported suicide attempt in adolescence and young adulthood

Within the QLSCD cohort, suicide attempt was measured at ages 13, 15, 17, 20 and 23. Adolescents were asked, *In the past 12 months, did you ever seriously think of attempting suicide?* and if so, *How many times did you attempt suicide?* dichotomised as no (never attempted suicide) or yes (≥1 suicide attempts). At ages 20 and 23, participants were asked if they *Ever went to the emergency department for a suicide attempt* and if they *Have ever been hospitalised for a suicide attempt*. Suicide attempt was then defined as reporting ≥1 suicide attempts (and/or self-reported hospitalisation/emergency visit for a suicide attempt) at any age between 13 and 23 years (coded as 1) or never reporting it (coded 0). Similarly, within the QLSKC cohort, structured interviews assessed suicide attempt at ages 15 and 22 using the Diagnostic Interview Schedule for Children and Adults, respectively.^25 26^ At age 15, participants and their parents were asked: *Have you/your child tried to kill yourself/themselves?*. The same question was assessed at age 22 to the participants only. A variable for lifetime suicide attempt was derived, coded 1 if the participant or his/her parent reported a suicide attempt at either age 15 or 22 years, and 0 if not. For QNTS, adolescents at age 19 were asked; *Have you ever attempted suicide?*, answered as no (0) or yes (1). For the purpose of this study, a suicide attempt was defined as an act in which a person harms themselves with the intention to die, and survives,^27^ distinct from self-harming behaviour with no intention to die. However, we acknowledge the difficulties to determine intentionality behind a self-harm behaviour,^28^ especially in youth and only based on self-reported answers to survey questions.

#### Covariates

The following covariates were a priori selected for the multivariable models based on the literature.^29 30^

Background, child and family characteristics: sex of the child; maternal age of the mother at time of first survey administration; highest education obtained by the mother when the child is 6 years old (a proxy for family socioeconomic status); family structure at age 6 (a categorical variable differentiating between intact, single parent and blended families); positive (eg, calming discussing a problem with the child) and harsh (eg, hitting the child when they were difficult) parenting behaviours at age 6 reported by the person most knowledgeable about the child (the mother in >95% of the cases) using the Strayhorn and Weidman’s Parent Practices Scale in the QLSCD,^31^ Emotional Climate for Children Scale^32^ in the QLSKC and the Parental Cognitions and Conduct Towards the Infant Scale in both QLSCD and QNTS.^33^ To harmonise these variables, they were Z-score transformed in each cohort, so that a 1-SD unit increase in the score would indicate higher levels of positive/harsh parenting behaviours with respect to the average of the specific cohort.

Baseline (age 6 years) mental health symptoms and ADHD medication use, internalising (eg, anxious/depressive) and externalising (eg, conduct problems) symptoms of the child, were measured with teacher-reported items from the SBQ; ADHD medication used during middle childhood was assessed by reports from the person most knowledgeable of the child at age 6, 7, 8, 10 and 12 in the QLSCD; 9, 10, 11, 12, 13 and 15 in the QLSKC; and 15 in the QNTS. We created a variable corresponding to any use of ADHD medication during the assessed period.

### Data analysis

#### Identifying childhood profiles of ADHD symptoms

We jointly estimated developmental trajectories of hyperactivity–impulsivity and inattention (ADHD) symptoms from 6 to 12 years of age using parallel process latent growth modelling in Mplus. This longitudinal data-driven analysis technique allowed us to joint model the trajectories of hyperactivity–impulsivity and inattention from age 6 to 12. It resulted in the identification of different profiles characterised by distinct developmental patterns of both hyperactivity–impulsivity and inattention. We estimated models with 1 to 6 latent profiles, and the selection of the best model was based on methodological considerations. These included need for the model to minimise the Bayesian Information Criterion, and the accuracy of the classification of the individuals in the different classes (entropy, ranging from 0 to 1, with 1 indicating perfect classifications) and the interpretability of the model. ADHD symptom profiles were derived on the whole sample in the main analysis, then on male and female subsamples separately as sensitivity analysis.

#### Longitudinal associations between childhood ADHD symptom profiles and youth suicide attempt

Associations between ADHD symptom profiles and suicide attempt were investigated using binary logistic regression, with robust SEs to account for the non-independence of the twins in QNTS. The model estimated the OR of reporting suicide attempt for each of the ADHD symptom profiles compared with the profile exhibiting the lowest level of symptoms. Given the probabilistic nature of the model classifying the participants in the different profiles, estimates were adjusted to take into account the uncertainty of classification using the predicted probabilities of class membership. We fitted three models with different adjustment levels: (1) only accounting for cohort effect, (2) further adjusted for sociodemographic and family characteristics, (3) further adjusted for baseline mental health symptoms and ADHD medication use. We ran multivariate multiple imputations with the Amelia package in R^34^ to account for missing data in the covariates, therefore models were estimated across 20 imputed datasets and then pooled. Models were fitted for the whole sample and separately for males and females. We a priori decided to estimate models for males and females separately. However, to statistically test sex differences, we also estimated the interaction between ADHD symptom profiles and sex on the additive scale and computed the interaction contrast ratio (ICR), quantifying the excess risk due to interaction, that is how much the combined effect of two factors (eg, a given profile of ADHD symptoms and female sex) exceeds the sum of their individual effects. Values >0 indicate positive interaction (ie, synergy: the joint effect of both factors exceeds the sum of their individual effects), values <0 indicate negative interaction (ie, antagonism: the joint effect is less than the sum of the individual effects) and 0 indicates no interaction (ie, the effects of the two factors are additive). We also calculated the attributable proportion (AP), quantifying the proportion of the combined (synergic/antagonist) effect of two factors that can be attributed to their interaction (as opposed to their independent effects), that in this context represents the proportion of the putative effect on the outcome due to the combination of a given ADHD symptom profile (compared with the profile with lowest symptoms) and female sex (compared with male sex).^35^

## Findings

**Figure 1:**
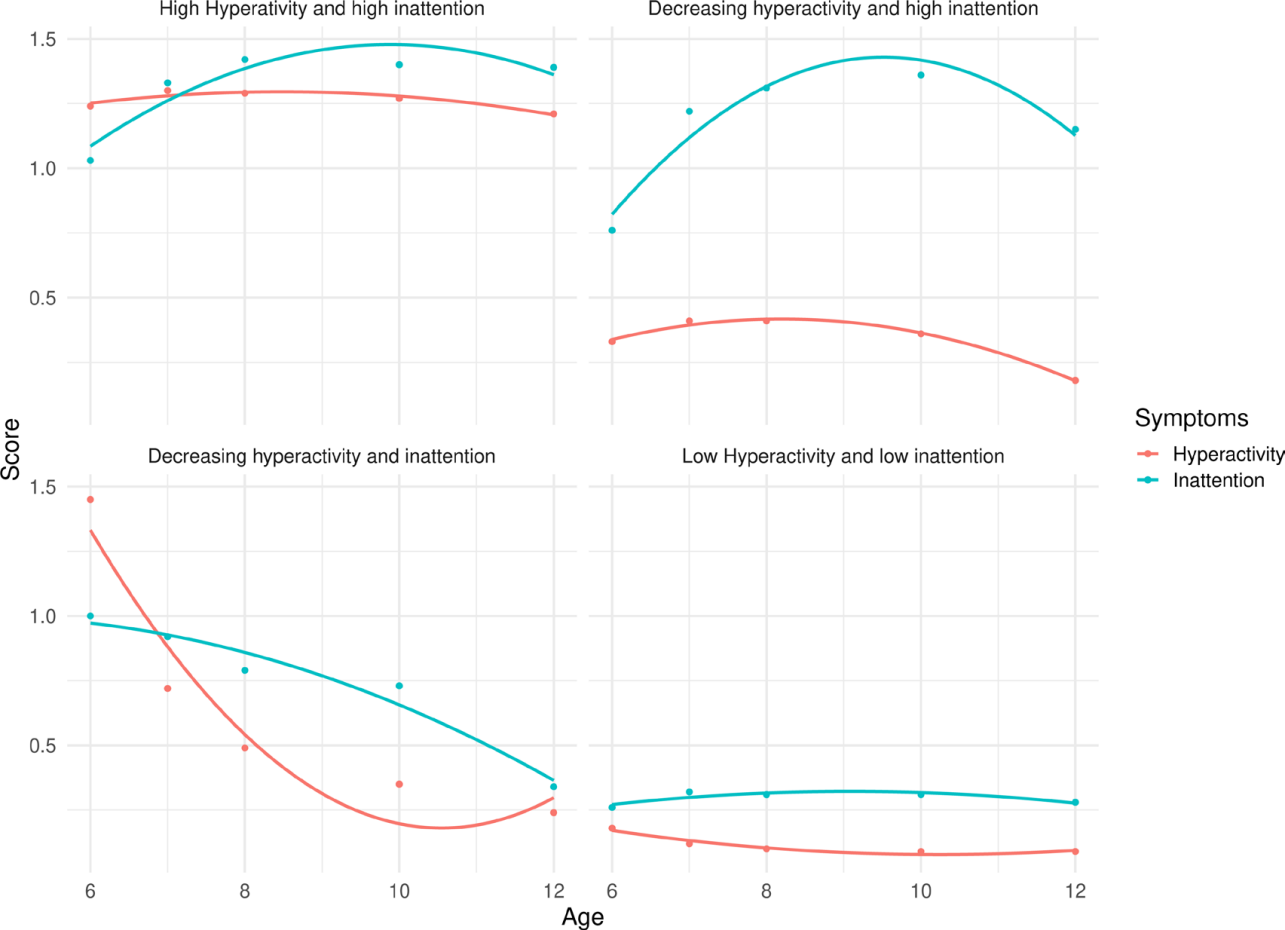
Profiles of ADHD symptoms. The figure shows the observed (dots) and estimated (lines) course of hyperactivity–impulsivity (red) and inattention (blue) symptoms (y-axis) over the course of middle childhood (x-axis) for the four identified ADHD symptom profiles. The model was estimated using Mplus V.8.11. We used 20 initial stage random starts and retained 4 for final stage optimisations. The best log-likelihood value was replicated two times. Data were for the Québec Longitudinal Study of Child Development and were compiled from the compiled from the final master file of the (1998–2023), Gouvernement du Québec, Institut de la statistique du Québec. ADHD, attention-deficit/hyperactivity disorder.

We included 4399 children who were followed up for 8–12 years, of which 2195 (49.9%) were male, and 2204 (50.1%) were female (table 1). The best model identified the following four profiles (figure 1 and online supplemental table S1): (1) *low hyperactivity–impulsivity and low inattention* (2475 (56.3%)), including participants with persistently low symptoms from 6 to 12 years of age; (2) *low hyperactivity–impulsivity and high inattention* (919 (20.9%)), including participants with low symptoms of hyperactivity–impulsivity but high inattention symptoms during the follow-up period; (3) *high hyperactivity–impulsivity and high inattention* (491 (11.2%)), including participants with persistently high symptoms of both hyperactivity–impulsivity and inattention and (4) *decreasing hyperactivity–impulsivity and decreasing inattention* (514 (11.7%)), including participants with initially high symptoms of both hyperactivity–impulsivity and inattention at the beginning of the follow-up, then decreased (especially for inattention) over middle childhood. Sensitivity analyses for males and females separately resulted in consistent classification of participants in the ADHD symptom profiles (online supplemental table S2). Characteristics of children belonging to each profile are reported in table 1, showing significant differences across ADHD symptom profiles on all the covariates, except for positive parenting (p=0.121), while table 2 shows the rate of suicide attempt for each ADHD symptom profile, stratified by sex. Females were more likely to report suicide attempt than males (n=238, 10.4% vs n=119, 5.6%, total n=357, 8.1%). Suicide attempt was reported by 128, 206 and 23 individuals in QLSCD, QLSKC and QNTS, respectively.

**Table 1:** Sociodemographic, family and clinical characteristics

	Overall sample	By ADHD symptom profile				
		Low hyperactivity–impulsivity and inattention	Low hyperactivity–impulsivity and high inattention	High hyperactivity–impulsivity and high inattention	Decreasing hyperactivity–impulsivity and inattention	P value
n	4399	2475 (56.3%)	919 (20.9%)	491 (11.2%)	514 (11.7%)	
Male sex, n (%)	2195 (49.9)	1044 (42.2)	497 (54.1)	349 (71.1)	305 (59.3)	<0.001
Cohort, n (%)						<0.001
QLSCD	1490 (33.9)	843 (34.1)	317 (34.5)	185 (37.7)	145 (28.2)	
QLSKC, representative sample	1460 (33.2)	923 (37.3)	265 (28.8)	121 (24.6)	151 (29.4)	
QLSKC, disruptive sample	674 (15.3)	291 (11.8)	139 (15.1)	105 (21.4)	139 (27.0)	
QNTS	775 (17.6)	418 (16.9)	198 (21.5)	80 (16.3)	79 (15.4)	
Maternal age, mean (SD)	28.33 (4.97)	28.61 (4.87)	28.20 (4.97)	27.39 (5.08)	28.13 (5.19)	<0.001
Low maternal education, n (%)	1693 (39.5)	842 (34.7)	428 (47.9)	205 (43.8)	218 (43.6)	<0.001
Positive parenting, z-score, mean (SD)	0.00 (1.00)	0.00 (0.99)	0.05 (1.01)	−0.09 (1.05)	−0.01 (0.96)	0.121
Harsh parenting, z-score, mean (SD)	0.00 (1.00)	−0.12 (0.97)	0.06 (1.02)	0.31 (1.06)	0.19 (0.96)	<0.001
Non-intact family (%)	1235 (33.3)	623 (29.3)	295 (38.5)	162 (41.2)	155 (36.6)	<0.001
Internalising symptoms at age 6, mean (SD)	2.04 (2.22)	1.57 (1.88)	2.62 (2.53)	2.90 (2.54)	2.45 (2.27)	<0.001
Conduct problems symptoms age 6, mean (SD)	1.14 (1.87)	0.60 (1.23)	1.31 (1.91)	2.91 (2.63)	1.81 (2.18)	<0.001
ADHD medication use, n (%)	275 (7.1)	42 (1.9)	78 (9.60)	114 (27.5)	41 (8.8)	<0.001

P values refer to the test of equality of proportions (or means) across the four ADHD symptom profiles.

Data were for the Québec Longitudinal Study of Child Development and were compiled from the compiled from the final master file of the (1998–2023), Gouvernement du Québec, Institut de la statistique du Québec.

ADHD, attention-deficit/hyperactivity disorder; QLSCD, Quebec Longitudinal Study of Child Development; QLSKC, Longitudinal Study of Kindergarten Children; QNTS, Quebec Newborn Twin Study.

**Table 2:** Association between ADHD symptom profiles and suicide attempt

	Suicide attempts, n (%)	Model 1	Model 2	Model 3
All				
Low hyperactivity–impulsivity and inattention	171 (6.9%)	1	1	1
High hyperactivity–impulsivity and inattention	49 (10.0%)	1.60 (1.12–2.29)	2.05 (1.39–3.04)	1.77 (1.16–2.70)
Low hyperactivity–impulsivity and high inattention	85 (9.2%)	1.63 (1.20–2.23)	1.85 (1.34–2.55)	1.67 (1.20–2.32)
Decreasing hyperactivity–impulsivity and inattention	52 (10.1%)	1.64 (1.11–2.42)	2.01 (1.34–3.00)	1.80 (1.20–2.71)
Females				
Low hyperactivity–impulsivity and inattention	134 (8.5%)	1	1	1
High hyperactivity–impulsivity and inattention	22 (20.0%)	3.50 (2.04–6.00)	2.79 (1.59–4.88)	2.54 (1.39–4.63)
Low hyperactivity–impulsivity and high inattention	57 (14.0%)	2.11 (1.44–3.08)	2.00 (1.36–2.95)	1.81 (1.21–2.70)
Decreasing hyperactivity–impulsivity and inattention	25 (13.4%)	1.69 (0.97–2.93)	1.54 (0.88–2.71)	1.38 (0.78–2.45)
Males				
Low hyperactivity–impulsivity and inattention	37 (4.2%)	1		
High hyperactivity–impulsivity and inattention	27 (7.1%)	1.86 (1.07–3.26)	1.63 (0.92–2.89)	1.31 (0.70–2.44)
Low hyperactivity–impulsivity and high inattention	28 (5.5%)	1.66 (0.93–2.96)	1.57 (0.87–2.83)	1.41 (0.78–2.56)
Decreasing hyperactivity–impulsivity and inattention	27 (8.2%)	2.63 (1.44–4.78)	2.45 (1.34–4.50)	2.23 (1.20–4.13)

Model 1 presents associations only adjusted for cohort membership, model 2 is further adjusted for sociodemographic and family characteristics, model 3 is further adjusted for internalising and conduct problems at age 6 and ADHD medication use. Models were adjusted to take into account the uncertainty of classification using the predicted probabilities of class membership, so that individuals are weighted so that those who are more accurately classified to their classes contribute more to the estimates than those classified less accurately.

Data were for the Québec Longitudinal Study of Child Development and were compiled from the compiled from the final master file of the (1998–2023), Gouvernement du Québec, Institut de la statistique du Québec.

ADHD, attention-deficit/hyperactivity disorder.

**Figure 2:**
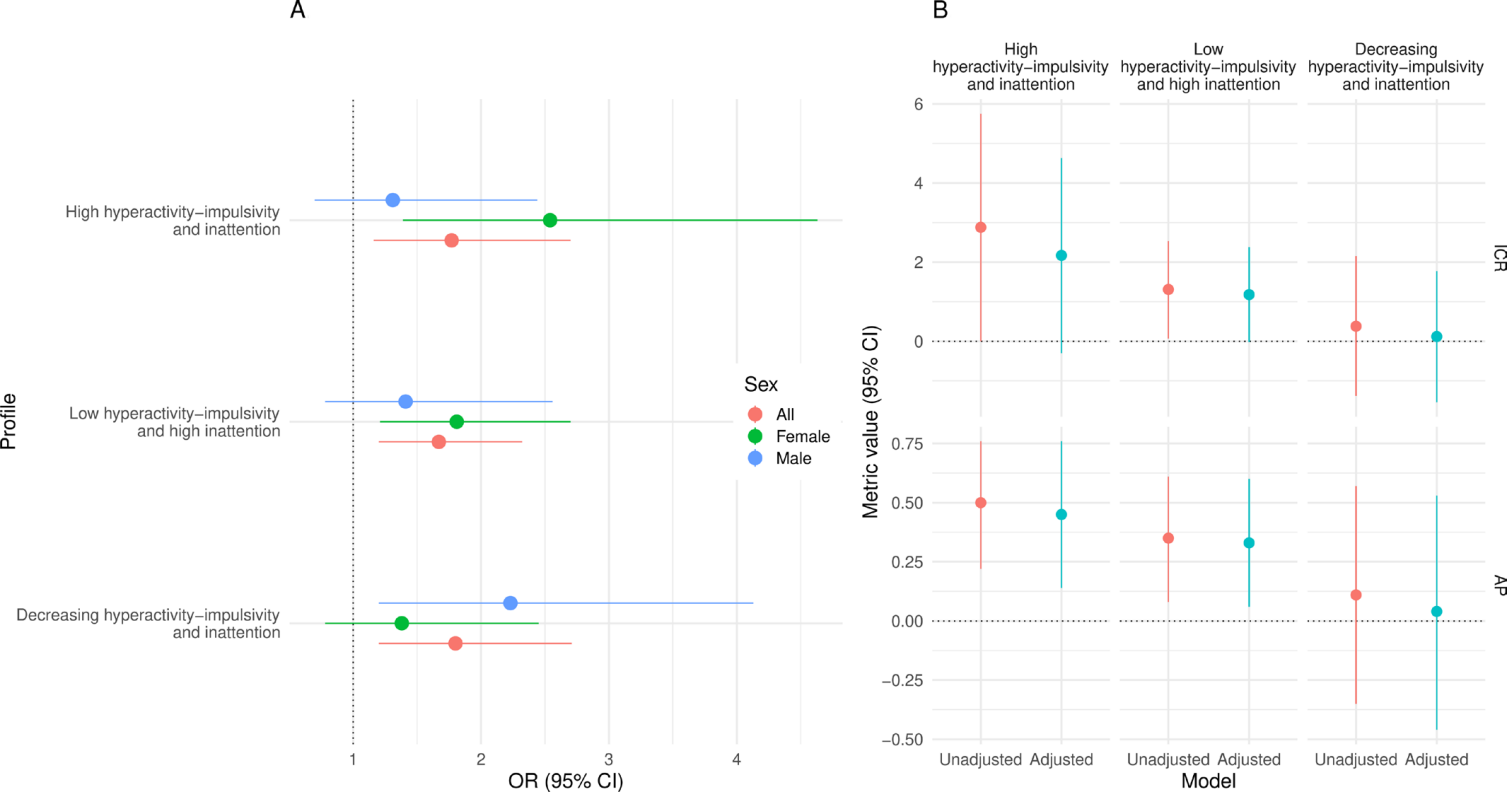
Adjusted estimates for the associations between ADHD symptom profiles and suicide attempt (**A**) and interaction metrics (**B**). Data were for the Québec Longitudinal Study of Child Development and were compiled from the compiled from the final master file of the (1998–2023), Gouvernement du Québec, Institut de la statistique du Québec. ADHD, attention-deficit/hyperactivity disorder.

Table 2 displays the OR (95% CI) for the association between ADHD symptom profiles and suicide attempt. Within the entire sample, the risk of suicide attempt was significantly higher in the *high hyperactivity–impulsivity and inattention* (OR: 1.77; 95% CI 1.16 to 2.70), low *hyperactivity and high inattention* (OR: 1.67; CI 1.20 to 2.32) and *decreasing hyperactivity–impulsivity and inattention* (OR: 1.80; CI 1.20 to 2.71) profiles compared with the *low hyperactivity–impulsivity and inattention* profile, even after adjusting for all the covariates (table 2, figure 2A). Interaction analyses suggested that risks of suicide attempt for females in the *high hyperactivity–impulsivity and high inattention* and *low hyperactivity–impulsivity and high inattention* profiles were significantly higher than for males, with ICR of 2.88 (CI 0.004 to 5.75, p=0.025; AP=0.42) and ICR of 1.30 (CI 0.07 to 2.53, p=0.019; AP=0.34). Although no clear evidence for an interaction was found for the *decreasing hyperactivity–impulsivity and inattention* profile, the ICR pointed towards a lower risk for females in this profile relative to males (0.38, CI −1.38 to 2.15, p=0.336; AP=0.11). Estimates were broadly similar for the adjusted model, although the CIs crossed the null, suggesting that covariates partially explain this interaction (figure 2B).

In analyses stratified by sex (table 2, figure 2A), compared with those in the *low hyperactivity–impulsivity and inattention* profile, the risk of suicide attempt was significantly higher for females in the *high hyperactivity and inattention* (OR: 2.54; CI 1.39 to 4.63) and *low hyperactivity–impulsivity and high inattention* (OR: 1.81; CI 1.21 to 2.7) profiles even after accounting for all the covariates, but not for females in the *Decreasing hyperactivity–impulsivity and inattention* profile (OR: 1.38; CI 0.78 to 2.45). Conversely, the risk of suicide attempt was significantly higher for males in the *Decreasing hyperactivity–impulsivity and inattention profile* (OR: 2.23; CI 1.20 to 4.13) even after accounting for all the covariates, but not for males in the *high hyperactivity–impulsivity and inattention* (OR: 1.31; CI 0.70 to 2.44) and in the *low hyperactivity and high inattention* (OR: 1.41; CI 0.78 to 2.56) profiles.

Sensitivity analyses using ADHD symptom profiles derived separately for males and females (online supplemental figure S2 and table S3), overall resulted in similar pattern of associations to the main analyses, in terms of direction of association, effect size and statistical significance. Furthermore, using a hard-coding classification of the individuals in the profiles instead of weighting for their estimated posterior probability (online supplemental table S4), yielded consistent results, with associations similar in direction, sizes and statistical significance to those found in the main analysis.

## Discussion

This large longitudinal population-based study explored the risk of suicide attempt in individuals with ADHD symptoms according to symptom profile and sex. We found that primary school children presenting with ADHD symptoms were at a higher risk of suicide attempt in adolescence and young adulthood, and that this association varied according to both sex and profile of ADHD symptoms. Specifically, females with a symptom profile characterised by elevated inattention symptoms, with or without hyperactivity-inattention symptoms, were at higher risk of suicide attempt by young adulthood than those with low symptoms, while no higher risk was found for males with similar symptom profiles. Among males, only those with a symptom profile characterised by initially high but then decreasing hyperactivity–impulsivity and inattention symptoms across middle childhood were at higher risk of subsequent suicide attempt.

We identified four distinct ADHD symptom profiles in the population in line with clinical literature based on diagnostic interviews and clinical assessments.^36^ Contrary to some previous studies, we did not find a group characterised solely by high hyperactivity–impulsivity symptoms. However, such a symptom profile has not been consistently identified in previous studies based on data-driven approaches.^37^ Furthermore, prior research mostly used cross-sectional designs or focused on relatively narrow development periods, in contrast with our longitudinal approach spanning 6 years of data collection. This allowed us to capture patterns of symptoms over a long period, accounting for developmental changes. Importantly, our study is the first to model the longitudinal course of hyperactivity–impulsivity and inattention symptoms jointly in the same statistical model. The higher proportion of males in the profile with high hyperactivity–impulsivity and inattention symptoms, and of females in the profile with high inattentive symptoms, is consistent with prior literature, including studies focusing on diagnoses of ADHD in clinical populations.^38^,^40^

Consistent with the previous studies,^5^,^9^ we found that children with high symptoms of hyperactivity–impulsivity and/or inattention were at higher risk of suicide attempt regardless of the specific symptom presentation and sex. However, by disentangling the heterogeneity in both symptom presentation and sex, this study uncovered important differences in suicide attempt risk in children with ADHD symptoms. These differences would have gone unnoticed if we had overlooked these sources of heterogeneity. Specifically, we found that for females, only those with symptom profiles characterised by the presence of high inattention (ie, the high hyperactivity–impulsivity and inattention and the high inattention profiles) had a higher risk of suicide attempt compared with those with low symptoms, while no higher risk was found for males with these same patterns of symptoms. A possible mechanism for this higher risk in females compared with male may imply the emergence of adolescent depressive symptoms. Studies have shown that patterns of comorbidity between ADHD and other symptoms differ by sex, with females being more prone to develop depressive symptom.^41^ This is particularly the case for profiles of ADHD symptoms characterised by high inattention, most common in females.^12^ Thus, female in our sample may have developed depressive symptoms in adolescence, which may have acted as more proximal risk factors for suicide attempt.^42^ Future studies are needed to confirm this possible explanation.

For males, the only profile associated with an increased risk of suicide attempt was the decreasing symptom profile. This profile may cluster children whose hyperactive and impulsive behaviour in early childhood are prodromic of later conduct or emotional regulation problems^43 44^—rather than ADHD itself—which in turn may have increased their risk of suicide attempt.^6^ This is in line with other studies that have shown more important comorbidity between ADHD and conduct problems in males than females^45^ as well as findings suggesting that hyperactivity in males precedes later conduct problems.^46 47^ Empirically testing these mechanisms in future studies is critical to pave the way to new potential avenues for suicide prevention. Future studies using mediation analyses should test whether symptoms of conduct, emotional regulation or other problems (including anxiety and autistic traits, often becoming apparent after the hyperactive behaviour has decreased with age or medication) mediate the association between this ADHD symptom profile and later risk of suicide attempt.

### Limitations

First, this study relied on teachers’ dimensional assessment of ADHD symptoms and not on clinical assessments. This implies that we cannot directly generalise our findings to clinical populations (eg, children with an established diagnosis of ADHD), and that future studies should replicate our analysis using clinical samples to inform practice. However, dimensional assessment of symptoms is highly relevant in population-based studies, and teachers are considered objective evaluator since they can compare the child with several other children. Second, information on medication reported by parents was likely affected by recall bias. Official prescriptions data should be used in future studies. Third, as in all longitudinal studies, attrition affected the initial representativeness of the sample, limiting the generalisability of the finding to the population. Indeed, individuals more at risk (including those with high symptoms and socioeconomic risks) are also more likely to be lost to follow-up, thus our findings may not be generalisable to those individuals and are likely to be an underestimation of the real associations. Fourth, although we adjusted for general cognitive skills, specific measures of cognition (including cognitive delay) possibly underlying ADHD symptoms were not available in the cohorts, and thus we could not control for this aspect in a fine-grained manner. Fifth, our trajectory analysis modelled symptoms from ages 6–12 years, thus not covering the full length of adolescence, a critical period in which symptoms of ADHD may have significantly changed from earlier periods of life. Sixth, the assessment of suicide attempt varied across cohorts and may have resulted in missing some cases when the questions referring to the past 12 months were asked every 2 years (as in the QLSCD). This underestimation of suicide attempt events may have yielded conservative estimates of associations. Seventh, our data-driven strategy to derive ADHD symptom profiles would benefit from replication in independent samples, especially those with diverse sociodemographic characteristics. Finally, it is worth acknowledging that similar shapes of developmental patterns have been observed across a range of developmental psychopathology studies, raising the possibility that such patterns may partly reflect artefacts of the modelling approach. However, their recurring appearance across independent samples and domains also suggests that they may capture genuine heterogeneity in symptom persistence, remission or timing of onset. Thus, while caution is warranted in interpreting these patterns as discrete clinical subtypes, they may still offer meaningful insights into the developmental dynamics of ADHD symptoms and help guide future longitudinal and mechanistic research.

### Clinical implications

This study found that the association between ADHD symptoms and suicide attempt is heterogeneous and varies according to both child sex and ADHD symptom profiles. For females, this risk is associated with profiles characterised by high inattention symptoms (with or without hyperactivity–impulsivity symptoms), while for males this risk is only associated with profiles characterised by initially elevated ADHD symptoms that decrease over the course of middle childhood. Taking into account these differences in assessment and clinical care may help optimise suicide prevention for children with ADHD symptoms. For example, female children presenting with high inattention should be monitored for their risk of suicide attempt, even if they do not show high hyperactivity symptoms. Similarly, male children with high hyperactivity and inattention symptoms should be monitored for their suicide attempt risk even if these symptoms decrease over time. Finally, to provide effective clinical care and suicide preventive interventions for children with ADHD symptoms, it is necessary to develop a better understanding of the inner turmoil, emotional struggle and mental pain of these individuals,^48^ for example, with qualitative studies exploring individual perspectives of youth with such symptoms.^49^

## Supplementary material

10.1136/bmjment-2025-301725 online supplemental file 1

## Data Availability

Data may be obtained from a third party and are not publicly available.
