# Supplementary material for: Association of hyperactivity–impulsivity and inattention symptom profiles with suicide attempt: an 18-year population-based cohort study
Source: BMJ Ment Health. 2025 Jul 11;28(1):e301725. doi: 10.1136/bmjment-2025-301725 (PMC12248219; doi:10.1136/bmjment-2025-301725)
Supplement: online supplemental file 1 [file bmjment-28-1-s001.docx]

**Supplementary material**

Association of hyperactivity-impulsivity and inattention symptoms profiles with suicide attempt: A 18-year population-based cohort study

Michel Spodenkiewicz, Ayla Inja, Samuele Cortese, Cedric Galera, Isabelle Ouellet-Morin, Sylvana M Côté, Michel Boivin, Frank Vitaro, Mara Brengden, Ginette Dionne, Johanne Renaud, Richard E. Tremblay, Gustavo Turecki, Marie-Claude Geoffroy, Massimiliano Orri

**Table S1.** Fit indices of the multitrajectory model

| **Groups** | **Log-likelihood** | **n. of Parameters** | **AIC** | **BIC** | **Entropy** |
| --- | --- | --- | --- | --- | --- |
| 1 | -31711.466 | 21 | 63464.932 | 63599.104 |  |
| 2 | -27555.352 | 28 | 55166.705 | 55345.601 | 0.845 |
| 3 | -26337.382 | 35 | 52744.764 | 52968.384 | 0.830 |
| **4** | **-25831.490** | **42** | **51746.980** | **52015.324** | **0.828** |
| 5 | -25598.496 | 49 | 51294.991 | 51608.059 | 0.799 |
| 6 | -24854.685 | 56 | 49821.370 | 50179.161 | 0.826 |

Entropy values vary from 0 to 1, with higher values indicating better classification accuracy. AIC, Akaike Information Criterion; BIC, Bayesian Information Criterion

Data were for the Québec Longitudinal Study of Child Development were compiled from the compiled from the final master file of the (1998–2023), ©Gouvernement du Québec, Institut de la statistique du Québec.

**Table S2.** Comparison between profiles derived from males and females subsamples combined and separate

|  |  | Profiles derived using males and females subsamples separate | | | |
| --- | --- | --- | --- | --- | --- |
|  |  | Low hyperactivity and low inattention | Decreasing hyperactivity and inattention | High hyperactivity and high inattention | Low hyperactivity and high inattention |
| Profiles derived using males and females subsamples combined | Low hyperactivity and low inattention | **2391** | 66 | 5 | 13 |
|  | Decreasing hyperactivity and inattention | 57 | **798** | 30 | 34 |
|  | High hyperactivity and high inattention | 3 | 16 | **436** | 36 |
|  | Low hyperactivity and high inattention | 33 | 33 | 30 | **418** |

The table compare the categorization of participants in the 4 profiles when the profiles are estimated in the whole sample (rows) or separately in males and females (columns). The unweighted Kappa was 0.87 (weighted Kappa, 0.89), and the proportions of agreement for the 4 profiles were 0.996, 0.993, 0.980, and 0.974, suggesting that the classification of participants is virtually the same in the two approaches.

Data were for the Québec Longitudinal Study of Child Development were compiled from the compiled from the final master file of the (1998–2023), ©Gouvernement du Québec, Institut de la statistique du Québec.

**Table S3.** Associations estimated from profiles derived separately in females and males

|  |  | Model 1 | Model 2 | Model 3 |
| --- | --- | --- | --- | --- |
| All | High hyperactivity-impulsivity and inattention | 1.84 (1.25-2.71) | 1.99 (1.35-2.94) | 1.78 (1.19-2.67) |
|  | Low hyperactivity-impulsivity and high inattention | 1.68 (1.17-2.40) | 1.97 (1.35-2.90) | 1.69 (1.11-2.57) |
|  | Decreasing hyperactivity-impulsivity and inattention | 1.71 (1.25-2.34) | 1.86 (1.35-2.58) | 1.67 (1.20-2.34) |
|  |  |  |  |  |
| Females | High hyperactivity-impulsivity and inattention | 1.77 (1.07-2.90) | 1.64 (0.99-2.71) | 1.44 (0.85-2.44) |
|  | Low hyperactivity-impulsivity and high inattention | 2.99 (1.80-4.98) | 2.46 (1.45-4.16) | 2.24 (1.28-3.93) |
|  | Decreasing hyperactivity-impulsivity and inattention | 2.14 (1.45-3.14) | 2.02 (1.36-3.00) | 1.82 (1.21-2.73) |
|  |  |  |  |  |
| Males | High hyperactivity-impulsivity and inattention | 2.80 (1.48-5.29) | 2.62 (1.37-4.99) | 2.40 (1.25-4.61) |
|  | Low hyperactivity-impulsivity and high inattention | 1.87 (1.07-3.27) | 1.63 (0.92-2.90) | 1.25 (0.66-2.38) |
|  | Decreasing hyperactivity-impulsivity and inattention | 1.69 (0.96-3.00) | 1.61 (0.90-2.86) | 1.40 (0.77-2.54) |

Data were for the Québec Longitudinal Study of Child Development were compiled from the compiled from the final master file of the (1998–2023), ©Gouvernement du Québec, Institut de la statistique du Québec.

**Table S4.** Association between ADHD symptoms profiles and suicide attempt using hard-coding classification of individuals in the most likely profile

|  |  | Model 1 | Model 2 | Model 3 |
| --- | --- | --- | --- | --- |
| All | High hyperactivity-impulsivity and inattention | 1.51 (1.08-2.11) | 1.86 (1.29-2.68) | 1.50 (1.00-2.25) |
|  | Low hyperactivity-impulsivity and high inattention | 1.41 (1.07-1.85) | 1.56 (1.18-2.07) | 1.38 (1.03-1.84) |
|  | Decreasing hyperactivity-impulsivity and inattention | 1.59 (1.14-2.20) | 1.85 (1.32-2.60) | 1.61 (1.13-2.29) |
|  |  |  |  |  |
| Females | High hyperactivity-impulsivity and inattention | 2.85 (1.72-4.71) | 2.26 (1.34-3.81) | 1.92 (1.07-3.44) |
|  | Low hyperactivity-impulsivity and high inattention | 1.82 (1.31-2.54) | 1.71 (1.22-2.40) | 1.49 (1.05-2.13) |
|  | Decreasing hyperactivity-impulsivity and inattention | 1.76 (1.11-2.78) | 1.59 (1.00-2.53) | 1.34 (0.82-2.18) |
|  |  |  |  |  |
| Males | High hyperactivity-impulsivity and inattention | 1.77 (1.06-2.95) | 1.57 (0.93-2.66) | 1.19 (0.66-2.15) |
|  | Low hyperactivity-impulsivity and high inattention | 1.37 (0.83-2.27) | 1.30 (0.78-2.17) | 1.15 (0.68-1.94) |
|  | Decreasing hyperactivity-impulsivity and inattention | 2.17 (1.30-3.64) | 2.05 (1.21-3.45) | 1.81 (1.06-3.10) |

Model 1 presents associations only adjusted for cohort membership, model 2 is further adjusted for sociodemographic and family characteristics, model 3 is further adjusted for internalizing and conduct problems at age 6 and ADHD medication use.

Data were for the Québec Longitudinal Study of Child Development were compiled from the compiled from the final master file of the (1998–2023), ©Gouvernement du Québec, Institut de la statistique du Québec.

**Figure S1.** Flowcharts of included participants


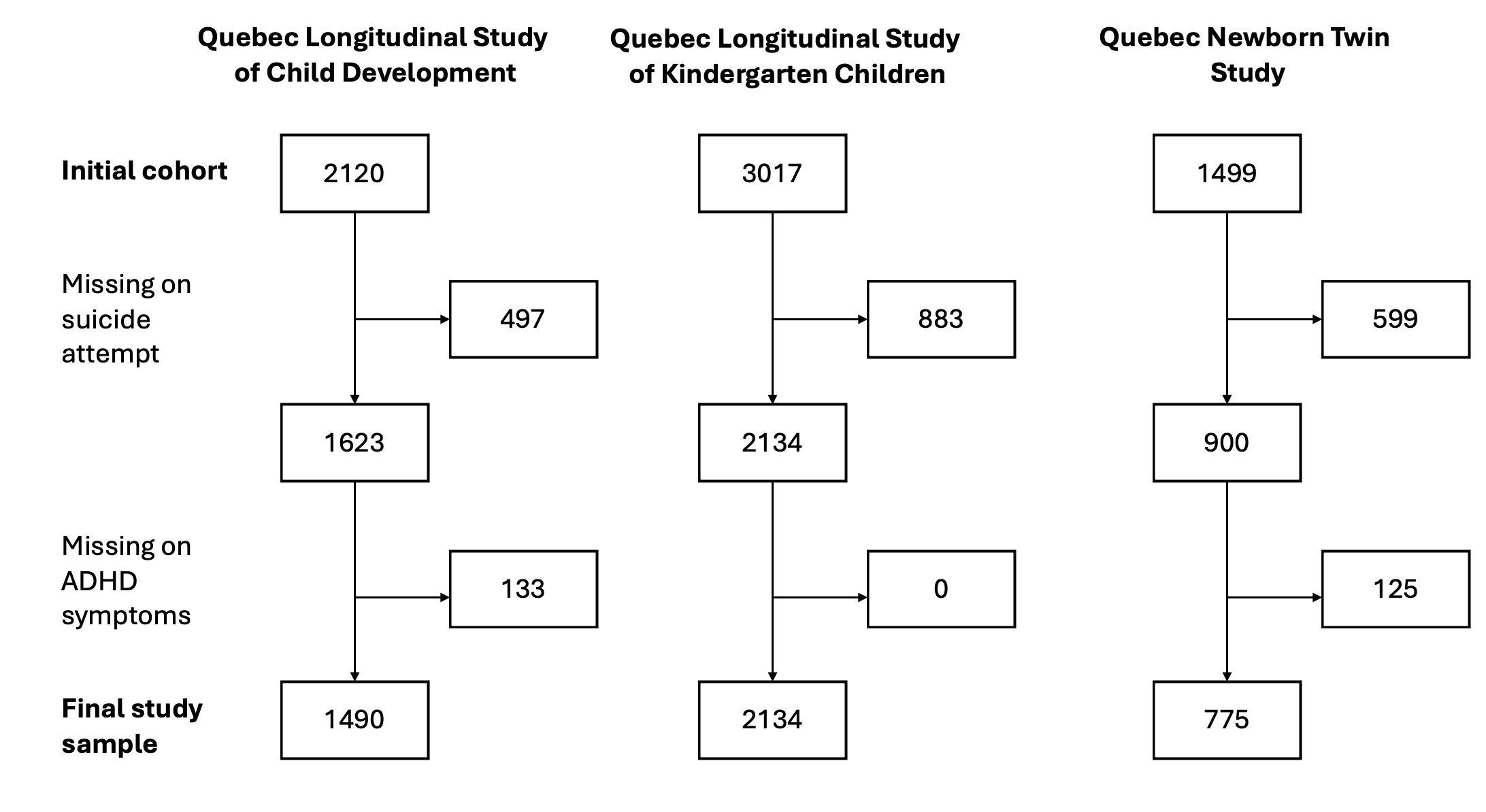


**Figure S2.** ADHD symptoms profiles derived separately from males and females


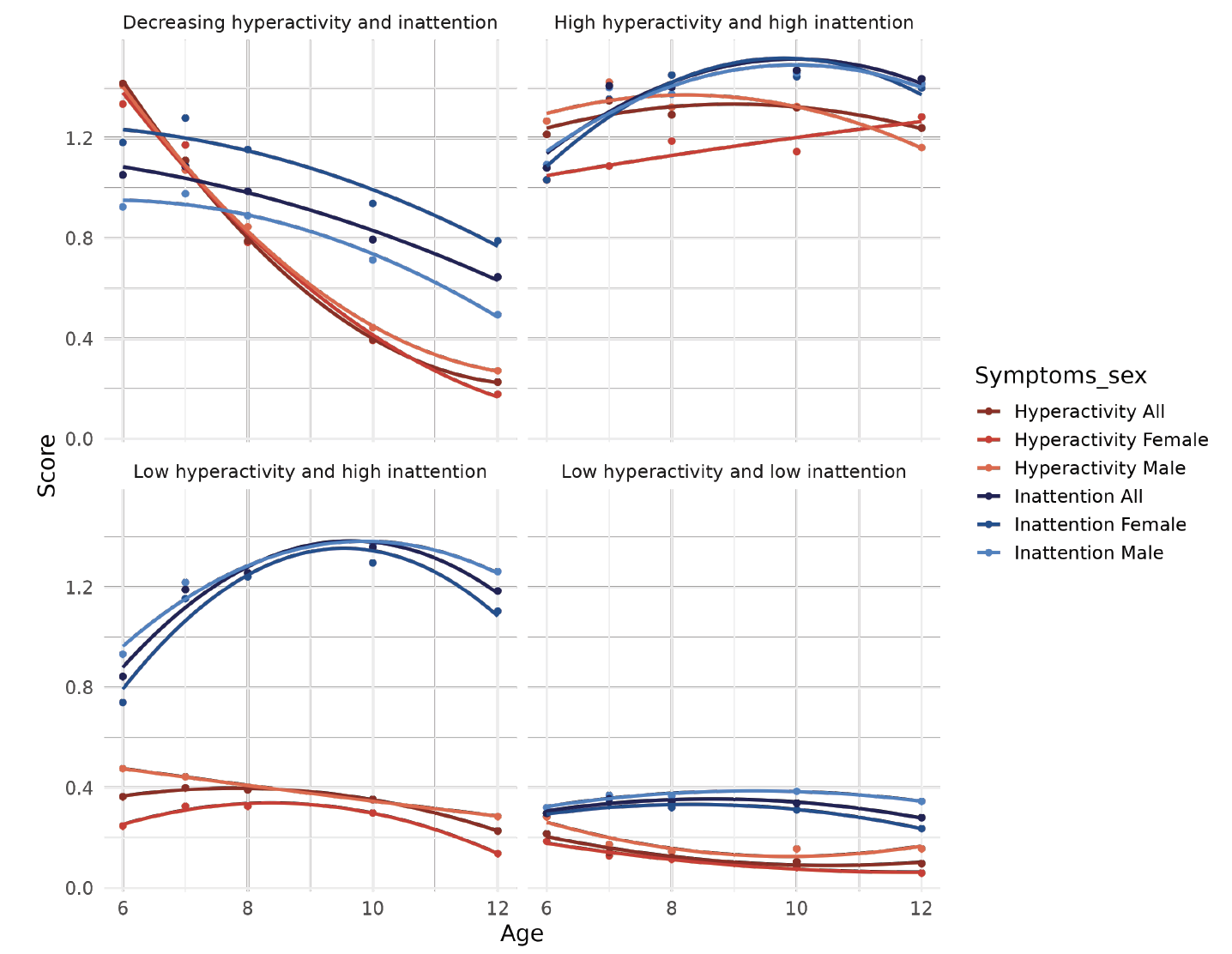


In the primary analysis, profiles were derived on the whole sample (sex combined). In sensitivity analyses, profiles were derived for the subsamples of males and females separately. The figure shows the profiles derived in males and females separately, as well as those derived on the whole sample.

Data were for the Québec Longitudinal Study of Child Development were compiled from the compiled from the final master file of the (1998–2023), ©Gouvernement du Québec, Institut de la statistique du Québec.
